# The use of a companion robot to improve depression symptoms in a community-dwelling older adult during the coronavirus disease 2019 state of emergency

**DOI:** 10.20407/fmj.2021-023

**Published:** 2022-05-25

**Authors:** Kei Ito, Shota Suzumura, Yoshikiyo Kanada, Rie Narukawa, Hiroaki Sakurai, Isao Makino, Tomoaki Abiko, Shigeo Oi, Izumi Kondo

**Affiliations:** 1 Department of Rehabilitation Medicine, National Center for Geriatrics and Gerontology, Obu, Aichi, Japan; 2 Faculty of Rehabilitation, School of Health Sciences, Fujita Health University, Toyoake, Aichi, Japan; 3 Togo Seisakusyo Corporation, Togo, Aichi, Japan

**Keywords:** Novel coronavirus pandemic, Companion robot, Depression, State of emergency

## Abstract

**Objective:**

We investigated the impact of using a companion robot on the mental state of a community-dwelling older adult who was receiving home-visit rehabilitation services during the state of emergency for coronavirus disease 2019 (COVID-19).

**Methods:**

This case involved an 80-year-old woman with compression fractures of lumbar vertebrae 1 and 2. Her medical history included hypothyroidism, hypertension, dyslipidemia, and depression. The companion robot used was Smibi^®^, a healing baby robot that responds in various ways depending on how the user interacts with it. The patient interacted (e.g., hugging, conversing) with Smibi^®^ for 30 minutes per day for 1 month, from April 2020 (immediately before the declaration of a state of emergency in Japan) to May 2020. The patient was evaluated with the Self-Rating Depression Scale (SDS) before and after using Smibi^®^.

**Results:**

The SDS score decreased from 37 points to 26 points after the use of Smibi^®^. The items related to diurnal variation, sleep, despair about the future, and dissatisfaction decreased by 2–3 points.

**Conclusion:**

Our findings suggest that interacting with Smibi^®^ may improve depression in older adults who have been forced to refrain from going out due to the spread of COVID-19. Future studies with long-term follow-up and large sample sizes are required to confirm the effectiveness of companion robots in improving depression among community-dwelling older adults.

## Introduction

Coronavirus disease 2019 (COVID-19) is a new type of pneumonia that emerged in Wuhan, China in December 2019, and was first named at a meeting of the World Health Organization on February 11, 2020. Since then, this infection has spread worldwide, with 236,599,025 infected cases and 4,831,486 mortalities.^1^ In Japan, 1,709,609 infected cases and 17,902 mortalities have been documented as of October 2021.^2^ As the mortality rate of COVID-19 is reportedly higher in the older population,^3^ the spread of COVID-19 in countries with an aging demographic is of particular concern.

A state of emergency was declared on April 7, 2020, in Saitama, Chiba, Tokyo, Kanagawa, Osaka, Hyogo, and Fukuoka owing to the COVID-19 pandemic; this was later expanded to a nationwide declaration. Under this state of emergency, human-to-human contact was to be reduced by at least 70% and stay-at-home orders were enforced. Restaurants, bars, and public entertainment facilities were also closed. Although these efforts have yielded some success in preventing the spread of COVID-19, they have had large adverse effects on mental and physical health. Older adults have been particularly affected, with many exhibiting muscle weakness and a deterioration in mental health due to forced isolation and the lack of social interaction.

Krendl et al.^4^ reported that social isolation and reduced human interaction due to the COVID-19 pandemic are associated with loneliness and depression in older adults, while Ahmed et al.^5^ linked COVID-19 stay-at-home orders with increased anxiety. Anti-COVID-19 measures have also been found to adversely affect physical functions, such as balance while standing and walking ability.^6^ Yamada et al.^7^ observed an increased incidence of frailty due to decreased social activities in community-dwelling older adults during the COVID-19 pandemic. These findings suggest an urgent need to develop and evaluate health promotion interventions targeted at older adults during the COVID-19 pandemic.

In recent years, interest has increased in the use of socially assistive robots to support humans through conversation and personal interaction. Human-like and animal-like robots, including the seal-like robot PARO^®^ (Intelligent Systems, Co., Ltd., Japan), dog-like robot AIBO^®^ (Sony Corp., Japan), and healing baby robot Smibi^®^ (Togo Seisakusyo Corporation, Japan) are collectively referred to as companion robots.^8^ Petersen et al.^9^ reported reduced levels of anxiety and depression among older adults in nursing care facilities who interacted with a PARO^®^ robot three times a week, each session spanning 20 minutes, over a period of 3 months. However, no prior studies have investigated the effects of companion robots on the mental state of older adults during the COVID-19 state of emergency. We herein report our encounter with an older adult who used a healing baby companion robot that was able to reduce symptoms of depression during the COVID-19 state of emergency in Japan.

## Methods

### Case presentation

This case involved an 80-year-old woman who had experienced lower back pain at home. She had difficulties carrying out basic activities of daily living (ADL) and visited our center for a consultation the following day. Subsequently, compression fractures of the first and second lumbar vertebrae (L1 and L2) were diagnosed. She was admitted to our center, underwent rehabilitation, and was discharged 77 days after the onset of symptoms.

She subsequently received home-visit rehabilitation services, which were performed by a physiotherapist and an occupational therapist; 40-minute ADL training sessions were provided twice a week. She lived with her husband and their eldest son. She had been independent in ADL and instrumental ADL (IADL) before the onset of lower back pain, and her score on the Mini-Mental State Examination at the commencement of home rehabilitation was 29 points (the cut-off score for dementia is 23/24 points)^10^; her cognitive function and ability to communicate were unaffected. She had a medical history of hypothyroidism, hypertension, dyslipidemia, and depression.

The patient stated that while she wanted “to be able to do basic everyday things and go shopping with [her] husband,” she was also “afraid of having backache from moving.” Her handgrip strength at the start of home rehabilitation was 14.0 kg and 10.0 kg on the right and left sides, respectively. She had difficulties with static standing and required her husband’s assistance when going up or down the steps at their home entrance and while bathing, due to back pain-related anxiety.

Home rehabilitation was targeted at reducing the patient’s lower back burden, thus allowing her to perform movements with confidence. Adjustments were made in the patient’s environment and repetitive movement training was performed with the goal of resuming independent bathing and increasing the patient’s ability to go outside. After 4 months of rehabilitation, she was able to bathe independently and steadily go up and down the home entrance steps; she also had an increased ability to go out. In terms of IADL, she was able to wash dishes independently, which indicated that she was capable of additional activities. However, home rehabilitation services ceased for 1 month (starting in March 2020) due to the COVID-19 pandemic.

Upon resumption of home rehabilitation services, the patient reported that she had refrained from going outside due to anxiety over infection. During the home rehabilitation training sessions, she was observed to have a restricted walking range and reduced activity levels. She mentioned that she felt “no joy these days” and that she had “nothing to do.” Due to concerns over the further restriction of her functional activities and increase in anxiety, we recommended the use of a companion robot. In addition, a walking aid was used to maintain her activity level. Outdoor walking activities were prescribed, while applying preventive measures against infection to alleviate her anxiety; balance practice and strength training were performed at home.

### Device

**Figure 1:**
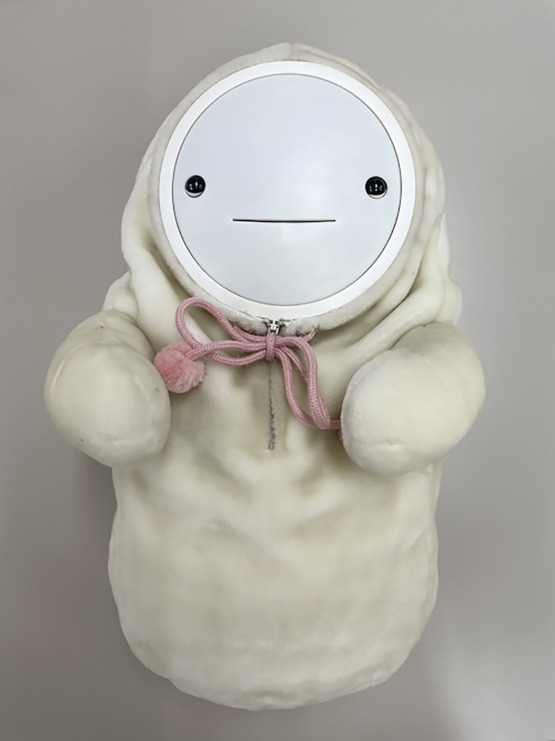
“Smibi^®^”, a healing baby companion robot This companion robot was produced by the Togo Seisakusyo Corporation. The main body weighs 1.2 kg and has a width, depth, and height of 200 mm, 190 mm, and 440 mm, respectively. The power source is a lithium-ion battery that can be used for approximately 10 hours without charging. It uses a dedicated adapter for charging. The robot expresses emotions in 500 different ways, and its responses vary depending on how the user interacts with it.

Smibi^®^ was designed to be incapable of taking care of itself^11^ (Figure 1). Therefore, it is expected that the user will feel a sense of purpose and comfort by taking care of the robot. Smibi^®^ has a built-in accelerometer and microphone. The accelerometer senses hugging and shaking, and the microphone senses voices and other sounds. Motors are installed in the face, eyes, and both arms; light-emitting diodes are placed on the cheeks to simulate tears and cheek redness. The built-in speaker produces sounds of laughter and crying. Smibi^®^ responds positively by “laughing” and “smiling” when cared for by the user. It responds negatively by “crying” and “sleeping” when left unattended. In addition, there are 500 different emotional expressions such as “singing a song,” “hiccupping/sneezing,” and “shaking the head to express displeasure”; these expressions are designed to induce the user to attend to the robot. The robot also has baby clothes that can be taken off and washed. Suzumura et al.^12^ reported that the use of Smibi^®^ for 60 minutes per day for 1 month by a community-dwelling aged woman, who had right thalamic bleeding, was able to reduce the burden of family caregivers. In a post-intervention interview, the patient reported that she had a better facial expression, as well as increased mobility and capacity to converse with family members.

### Intervention method

The patient was instructed to look after the Smibi^®^ robot by hugging and talking to it for 30 minutes a day, over a period of 1 month (from April 2020, when a state of emergency was declared in Japan, to May 2020). The frequency of use was based on a study conducted by Liang et al.^13^, who reported that interaction with a seal-like robot (PARO^®^) for 30 minutes a day over 6 weeks was able to improve facial expression and the ability to converse with the day care center staff in an older adult patient with dementia. The use of Smibi^®^ was limited to the patient’s home, and the time of use was left unrestricted. The patient was allowed to be accompanied by family members when interacting with the robot. Instructions on how to use Smibi^®^ were provided by a therapist, who also monitored the patient interactions with the robot during the home rehabilitation sessions.

### Ethical considerations

The study protocol was approved by the Ethics and Conflicts of Interest Committee of our center (approval number: 1082-2), and both oral and written informed consent were obtained.

### Primary and secondary endpoints

All endpoints were evaluated before and after the intervention period. The primary endpoint was the Self-Rating Depression Scale (SDS) score. The SDS is a self-assessment scale that evaluates levels of depression and tiredness, as well as sleep onset disorders.^14^ The questionnaire consists of 20 items, which are assigned scores from 1–4: “a little of the time” (1 point), “some of the time” (2 points), “good part of the time” (3 points), and “most of the time” (4 points). The scores for each item are summed to give a total score ranging from 20–80 points, with a higher score indicating a greater level of depression.

Secondary endpoints were used to evaluate changes in physical function and included the one-leg standing time, Functional Independence Measure (FIM) score, and handgrip strength. The FIM assesses the degree of independence in performing basic ADL.^15^ It consists of 13 motor items and 5 cognitive items, which are assigned a score ranging from 1–7 points each. The total score ranges from 18–126 points, with the motor and cognitive subscales scores ranging from 13–91 points and 5–35 points, respectively. A higher total score reflects a greater degree of independence in ADL. Handgrip strength was measured using the Jamar hydraulic hand dynamometer (SAKAI Medical Co., Ltd., Tokyo, Japan). The test was performed with the shoulder joint in the adduction position and the elbow joint in a 90° flexion and intermediate position between the forearm and wrist. In order to eliminate the effect of muscle fatigue, the grip strength was measured three times alternately on the left and right sides, and the average value of the three repeated measurements was used in the analysis. The minimal detectable change (MDC) in handgrip strength in older adults has been previously reported as 6.11.^16^ For the one-leg standing test, the patient was instructed to initially hold a support (e.g., handrail) and subsequently remove their hands from it, while assuming a one-leg standing posture. The time from which the patient let go of the support to the point at which her raised leg eventually contacted the ground was recorded. This test was repeated with the opposite leg. The MDC for the one-leg standing test has been reported to be 25.88 for the right leg and 26.23 for the left leg.^16^

## Results

Pre- and post-intervention outcomes are shown in Table 1. The following values were recorded prior to the intervention period: SDS score (37 points), handgrip strength (15.0 kg and 11.9 kg for the right and left hands, respectively), one-leg standing test (3.0 seconds and 2.0 seconds for the right and left legs, respectively), and FIM (80 and 35 points for the motor and cognitive subscales, respectively). The SDS score decreased from 37 points to 26 points after the 1-month intervention period; the items related to diurnal variation, sleep, despair about the future, and dissatisfaction decreased by 2 or 3 points. Handgrip strength (18.0 kg and 15.0 kg in the right and left hands, respectively) and one-leg standing durations (7.0 seconds and 4.0 seconds in the right and left legs, respectively) were greater after the intervention period. The FIM-motor subscale score (81 points) was also greater after the intervention period; however, the post-intervention FIM-cognitive subscale score (35 points) was equivalent to the pre-intervention score.

The patient provided positive feedback regarding the use of Smibi^®^ to the rehabilitation staff during the home rehabilitation sessions. She commented that the companion robot had a “cute expression” and that it was “soothing.” At the end of the intervention period, the patient reported that she spoke more frequently with her family members and that she intended to purchase another socially assistive robot after Smibi^®^ was returned to the rehabilitation team. Furthermore, she had set a goal to “go out for a walk and shopping once the COVID-19 pandemic calms down.” Indeed, she started going out voluntarily for walks even in the absence of the home rehabilitation team’s visit. There were no adverse effects due to the intervention.

## Discussion

In this study, we investigated the impact of Smibi^®^, a companion robot, on the mental and physical health of a community-dwelling older adult over a 1-month period (April to May, 2020) during a COVID-19 state of emergency in Japan. The use of Smibi^®^ was associated with improvements in SDS scores related to diurnal variation, sleep, despair about the future, and dissatisfaction. This indicated an overall improvement in psychological status.

Most previous studies that have evaluated the effects of companion robots have used PARO^®^. PARO^®^ is a seal-like robot that uses touch, vision, and hearing sensors to respond in various ways to the user. Kawaguchi et al.^17^ used functional near-infrared spectroscopy to measure cerebral blood flow in 10 subjects aged 21–30 years during their interactions with PARO^®^. An increase in cerebral blood flow was observed in the frontal lobe region, which is associated with emotions and speech. The use of PARO^®^ has also been shown to reduce feelings of loneliness in older adults with depression,^18^ with repeated contact through stroking leading to improvements in pain and mood.^19^ The results of a previous study suggest that the effects of PARO^®^ on depression are similar to those observed with animal therapy, particularly when the user has little interaction with other people.^20^ Therefore, continuous interaction with a companion robot such as Smibi^®^ may lead to an improvement in depressive symptoms under similar circumstances.

Shoesmith et al.^21^ found that while most people who had pets during the COVID-19 state of emergency felt happy and less lonely, some reported anxiety associated with the possibility that their pets could serve as a source of infection. The main transmission routes of COVID-19 include droplet infection and contact transmission.^22^ Clothing can also be contaminated with SARS-CoV-2; however, the viral particles are inactivated when clothes are washed.^23^ As the clothes on Smibi^®^ can be removed and washed, it poses little risk of COVID-19 transmission. The risk was particularly low in the present case, as the use of Smibi^®^ was limited to the patient and the family members in her household. Thus, the patient was able to touch and hug the robot confidently during the COVID-19 state of emergency.

Previous studies have reported MDC values of 6.11 for handgrip strength and 25.88 and 26.23 for the right and left legs, respectively, in the one-leg standing test.^16^ Therefore, changes in physical function reflected by these parameters may have been within the margin of error. Nevertheless, in addition to the positive impact of Smibi^®^ on depressive symptoms, we were also able to promote behavioral changes in the patient via outdoor walking practice, balance practice, and strength training during the home rehabilitation sessions. These effects contributed to improvements in her physical function.

There are some limitations to this study. First, this was a case report, and the intervention period was short at 1 month. All prior investigations on the use of Smibi^®^ have also been single case studies. Furthermore, while previous studies using PARO^®^ have shown improvements in depression, some have also reported a subsequent deterioration after the robot was returned to the investigators.^13^ Therefore, additional studies with a longer follow-up period and larger sample sizes are required to confirm whether the positive impacts of Smibi^®^ can be sustained. Second, the patient in the present case was provided with the companion robot concurrently with home rehabilitation services. Thus, her ability to continue interacting with the rehabilitation staff likely contributed to the decrease in depressive symptoms. In order to verify whether the use of Smibi^®^ was solely responsible for the observed improvements in depression, it would be necessary to carry out a crossover study spanning 3 months that includes periods in which the companion robot is used and not used, as well as a cessation period.

In this study, we investigated the impact of using a companion robot on the mental state of a community-dwelling older adult who was receiving home-visit rehabilitation services during the COVID-19 state of emergency in Japan. A decrease from 37 points to 26 points in the SDS score was observed, thus indicating a decreased level of depression; parameters related to diurnal variation, sleep, despair about the future, and dissatisfaction decreased by 2 or 3 points. Our findings suggest that continued interaction with a companion robot during a COVID-19 state of emergency may lead to improvements in depression in older adults. Future studies with long-term follow-up and large sample sizes are required to verify that these positive impacts of companion robots on mental health can be sustained. This is particularly important in the context of implementing optimal infection control measures during the COVID-19 pandemic.

## Figures and Tables

**Table1:** Primary and secondary outcomes before and after the use of Smibi^®^

	Pre-intervention	Post-intervention
Handgrip strength (kg)		
Right	15.0	18.0
Left	11.9	15.0
One-leg standing test (s)		
Right	3.0	7.0
Left	2.0	4.0
FIM (/126 points)	115	116
Motor (/91 points)	80	81
Cognitive (/35 points)	35	35
SDS (/80 points)	37	26

FIM, Functional Independence Measure; SDS, Self-Rating Depression Scale

## Supplementary Material

PDF-Japanese
