# Supplementary material for: The use of a companion robot to improve depression symptoms in a community-dwelling older adult during the coronavirus disease 2019 state of emergency
Source: Fujita Med J. 2022 May 25;9(1):47–51. doi: 10.20407/fmj.2021-023 (PMC9923449; doi:10.20407/fmj.2021-023)
Supplement: Supplementary file 1 — PDF-Japanese [file fmj-9-047-s001.pdf]

新型コロナウイルス感染症の流行に伴う隔離期間中にコンパニオンロボットを使用し抑うつ性が改善した地域在住高齢者の一症例

ランニングタイトル : 新型コロナウイルス感染症による隔離期間中のコンパニオンロボットの使用効果

Kei Ito, RPT[1], Shota Suzumura, OTR, MA[1,2], Yoshikiyo Kanada, RPT, PhD[2], Rie Narukawa, OTR[1], Hiroaki Sakurai, RPT, PhD[2], Isao Makino[3], Tomoaki Abiko[3], Shigeo Oi[3], Izumi Kondo, MD, PhD[1]

1. Department of Rehabilitation Medicine, National Center for Geriatrics and Gerontology, Obu, Aichi, Japan
2. Faculty of Rehabilitation, School of Health Sciences, Fujita Health University, Toyoake, Aichi, Japan
3. Togo Seisakusyo Corporation, Togo, Aichi, Japan

Type: Case Study

Corresponding author: Shota Suzumura, OTR, MA

Faculty of Rehabilitation, School of Health Sciences, Fujita Health University, Toyoake, Aichi, Japan
Phone: +81-562-93-9000
E-mail: shota.suzumura@fujita-hu.ac.jp

要旨

目的:新型コロナウイルス感染症（coronavirus disease 2019: COVID-19）の流行に伴う隔離期間中，訪問リハビリテーションを利用している地域在住高齢者の一症例に対してコンパニオンロボットの使用が精神状態に与える影響を調査した.

方法: 症例は第 1，第２腰椎圧迫骨折と診断された 80 歳代の女性である．既往には甲状腺機能低下，高血圧症，脂質異常症，うつ病を認めた．コンパニオンロボットは，使用者のあやし方により様々な反応を示す癒し型赤ちゃんロボットの Smibi®を用いた．症例は，Smibi®のお世話（抱っこや会話など）を毎日 30 分，1 ヶ月間継続して行った．使用期間は，日本で緊急事態宣言が発令される直前の 2020 年 4 月から発令後の 2020 年 5 月の 1 ヶ月間とした．Smibi®の使用前と使用後に Self-rating Depression Scale（SDS）を評価した.

結果: 抑うつ状態の程度を表す SDS スコアは 37 点から 26 点に減少し，特に日内変動，睡眠，将来への絶望，不満に関する項目で 2，3 点減少した.

結論: Smibi®との交流で，COVID-19 の感染拡大に伴う外出自粛を強いられた高齢者の抑うつ性を改善する可能性が示唆された．今後は，コンパニオンロボットの効果を証明するため症例数を増やしていくとともに，持続的な効果を検証すべく長期的な検証を行う必要がある.

キーワード:　新型コロナウイルス感染症　コンパニオンロボット　抑うつ性　隔離

序論

新型コロナウイルス感染症（coronavirus disease 2019: COVID-19）は，2019 年 12 月に中国の武漢を発生源として出現した新型肺炎であり，2020 年 2 月 11 日に世界保健機関（World Health Organization: WHO）に命名された．世界各国にその影響が広まっており，世界では 2021 年 10 月時点で感染者 236,599,025 人，死者 4,831,486 人 [1]，日本では感染者 1,709,609 人，死者 17,902 人となった [2]．また，COVID-19 に罹患した患者の死亡率は，高齢者で高まることが報告されており [3]，高齢化が進む諸外国において COVID-19 の流行は，大きな問題となっている．

我が国では，2020 年 4 月 7 日に埼玉県，千葉県，東京都，神奈川県，大阪府，兵庫県，福岡県を対象に緊急事態宣言が初めて発令され，その後全都道府県に拡大した．緊急事態宣言の期間中は，人と人の接触を最低 7 割削減が求められ，外出の自粛，施設使用停止，飲食店の営業自粛などが要請された．これらの取り組みは，COVID-19 の感染拡大を予防することにおいて一定の成果を示しているが，社会的交流や外出機会の減少によって，高齢者の筋力低下，精神状態の悪化など心身機能に多大な影響を及ぼしている．

Krendl[4] は，COVID-19 の感染拡大による社会的孤立や人との関わりの減少は，高齢者の孤独感や抑うつに関与すると報告し，Ahmed[5] は，COVID-19 に伴う外出自粛の影響により人々の不安が増大したと報告した．また，Keir[6] は起立動作，立位バランス，歩行能力の低下など人々の身体機能に影響を与える可能性も報告している．Yamada[7] は，COVID-19 の流行に伴う地域在住高齢者の社会的活動の減少により，フレイル発生率が高くなったと報告した．これらの報告から，高齢者における COVID-19 の感染対策と健康の維持，増進のための具体的な支援を並行して検討することが重要な課題であるといえる．

近年，人との対話や支援を目的とした社会支援型ロボット（socially assistive robots: SAR）が注目を集めている．その中でもロボットの外観が人型や動物型であるものはコンパニオンロボットと総称されており [8]，アザラシ型ロボット PARO®（株式会社知能システム，日本），犬型ロボット AIBO®（ソニー株式会社，日本），癒し型赤ちゃんロボット Smibi®（株式会社東郷製作所，日本）など多くのコンパニオンロボットが提案されている．Petersen[9] は，施設入所中の高齢者に対して PARO®を週 3 回，20 分， 3 ヶ月間使用させたことにより，不安の軽減やうつ状態の改善を認めたと報告している．しかしながら，COVID-19 の流行に伴う隔離期間中

にコンパニオンロボットを使用して高齢者の精神状態に与える影響を調査した報告はない．今回，我々は緊急事態宣言による外出自粛期間中に癒し型赤ちゃんコンパニオンロボットを使用して，抑うつ性が改善した症例を経験したため報告する．

方法

症例紹介

症例は 80 歳代の女性である．自宅で腰痛が出現し，翌日に基本的日常生活動作（Activities of Daily Living: ADL）が困難となったため当センターを受診し第 1，第２腰椎圧迫骨折と診断された．当センターに入院し，リハビリテーションを受け，発症 77 日後に自宅退院となった．退院後は，理学療法士と作業療法士による訪問リハビリテーション（以下: 訪問リハ）を週 2 回，1 回 40 分実施し，ADL の練習を行っていた．家族構成は本人，夫，長男の 3 人暮らしであり，発症前の ADL，Instrumental Activities of Daily Living（IADL）は自立していた．訪問リハ開始時の Mini-Mental State Examination(MMSE)は 29 点であった．認知症の可能性を判断するカットオフ値は 23/24 点であり [10]，本症例の認知機能は保たれており，コミュニケーション能力は維持されていた．既往には，甲状腺機能低下，高血圧症，脂質異常症，うつ病を認めた．症例のニードは，「身の回りのことが出来るようになりたい，夫と買い物に出かけたい」であった．しかし，症例から「動くと腰痛になりそうで怖い」といった訴えが聞かれた．訪問リハ開始時の握力は右 14.0kg，左 10.0kg であり，静止立位は困難であった．ADL は，玄関の段差昇降や入浴動作で腰痛への不安から夫の介助が必要であった．訪問リハは，腰部の負担を減らして安心して動作が行えることを目的とし，入浴動作の自立と外出機会の獲得を目標として，環境調整と動作練習を繰り返し行った．開始 4 ヶ月後には入浴動作は自立し，段差昇降も安定し外出機会が増えた．IADL は，食器洗いを自身で行うようになり、活動の拡大を認めた．しかし，Covid-19 の流行に伴い，症例は 2020 年 3 月から 1 ヶ月間，訪問リハの利用を停止した．再開後の症例は，感染の不安から屋外歩行を自粛し，訪問リハ中も歩行範囲を自ら制限し活動の縮小を認めた．また「最近は楽しみがない」，「やることがない」といった発言が聞かれた．今後，更なる活動の縮小や不安感の増大が懸念され，コンパニオンロボットの使用に至った．介入期間中の訪問リハは，活動量を担保するため症例の歩行補助具を検討し，感染予防策を徹底して症例

の不安を取り除いたうえで屋外歩行練習を行った．その他に自宅内でのバランス練習や筋力トレーニングを行った．

機器紹介

Smibi®は，癒し型赤ちゃんロボットとして開発されたコンパニオンロボットである[11]（Figure1）．何も出来ないロボットをコンセプトに作成された．利用者がロボットの世話をすることで，生きがいや安らぎを感じられることを期待されている．Smibi®は，加速度センサーとマイクが内蔵されている．加速度センサーは，対象者の抱き方や揺れを感知し，マイクは声かけや音を感知する．両腕，顔面，目に各モーターが設置され，頬の部分には涙や頬の赤らみを表現するために LED が配置されている．またスピーカーの内蔵により笑い声や泣き声を発声する．これらの機能により Smibi®は，使用者にあやされると「笑う」，「微笑む」といったポジティブな反応を示し，使用者に放置されると「泣く」，「寝る」といったネガティブな反応を示す．その他に「歌を歌う」，「しゃっくり・くしゃみをする」，「首を振ってイヤイヤをする」など 500 通りの感情表現があり，使用者が思わずあやしたくなるように設計されている．着用しているベビー服は，着脱し洗濯することが可能である．Suzumura[12] は，右視床出血を呈した地域在住の女性高齢者に Smibi®を 1 日 60 分，1 ヶ月間使用した結果，家族の介護負担が軽減し，Smibi®使用後のインタビューでは，対象者の表情が豊かになったこと，家族間での会話の増加，座位時間の増加などポジティブな反応を示した．

介入方法

本研究では，症例は Smibi®のお世話（抱っこや会話など）を毎日 30 分，1 ヶ月間継続して行った．Liang[13] は，認知症高齢者にアザラシ型ロボット PARO®との 1 日 30 分間の接触を 6 週間継続させることで対象者の表情が改善し，スタッフとの会話が増加したことを報告した．この報告に従い，ロボットの使用時間を決定した．Smibi®の使用期間は，日本で緊急事態宣言が発令された 2020 年 4 月から 2020 年 5 月の 1 ヶ月間とした．Smibi®を使用する場所は自宅のみに限定し，使用する時間帯は統制せず，介入中は家族の付き添いを許容した．ロボットの使用方法の説明やロボットの使用状況の確認は，訪問リハ時に療法士が行った．

倫理的配慮

症例に対して，事前に口頭及び書面にて本介入の趣旨を十分に説明し，同意を得た．なお，本介入は当センターの倫理・利益相反委員会で承認を得ている（承認番号: 1082-2）．

主要評価項目および副次評価項目

Smibi®による介入前後に主要評価項目である Self-rating Depression Scale（SDS），副次評価項目である握力，片脚立位テスト，Functional Independence Measure（FIM）を評価した．SDS は，対象者の憂鬱感や疲れやすさ，入眠障害などの抑うつ性の程度を評価する自己評価式抑うつ尺度である[14]．20 項目の質問に分けられ，対象者は「ないかたまに」，「ときどき」，「かなりのあいだ」，「ほとんどいつも」の 4 件法で回答していく．点数は各項目 1 点から 4 点で採点され，合計 20 点から 80 点となる．点数が高ければ抑うつ傾向を示す．副次評価項目は，Smibi®の使用により症例の抑うつ性が改善されることが期待されるため，それに伴い症例の身体機能がどのように変化するかを把握するために行った．FIM は，基本的日常生活動作の自立度を評価する尺度である[15]．運動項目が 13 項目，認知項目が 5 項目から構成されている．各項目は 1 点から 7 点で検査者が採点する．運動項目は 13 点から 91 点，認知項目は 5 点から 35 点であり，合計点は 18 点から 126 点である．点数が高ければ高いほど，日常生活動作が自立していることを示す．握力の計測は，Jamar Hydraulic Hand Dynamometer（酒井医療株式会社製）を使用し，検査側の上肢は肩関節内転位，肘関節 90°屈曲位，前腕・手関節中間位とした．握力測定は筋疲労の影響をなくすために，左右交互に 3 回ずつ実施し，3 回の反復施行の平均値を用いた．高齢者における握力の Minimal detectable change（MDC）は，6.11 である[16]．片脚立位テストの計測は，症例に手すりなどの支持物に捕まらせ，片脚立位姿勢をとらせて支持物から両手が離れた時点で測定を開始し，挙上している側の下肢が地面に接するまでの時間を計測した．その後，反対側の下肢の計測を行った．片脚立位の MDC は，右下肢で 25.88，左下肢で 26.23 である[16]．

結果

本介入による有害な作用は認めなかった．Smibi®による介入前後の評価結果を Table 1 に示す．介入前の SDS は 37 点，握力は右上肢 15.0kg，左上肢 11.9kg，片脚立位テストは右脚 3.0 秒，左脚 2.0 秒，FIM は運動項目 80 点，認知項目 35 点であ

った．Smibi®使用から 1 ヶ月後の評価では，SDS は 37 点から 26 点に減少し，特に日内変動，睡眠，将来への絶望，不満に関する項目で 2 点から 3 点の減少となった．握力は右上肢 18.0kg，左上肢 15.0kg，片脚立位テストは右脚 7.0 秒，左脚 4.0 秒，FIM は運動項目 81 点，認知項目 35 点であった．介入開始後より，訪問リハでスタッフが自宅に伺った際，症例自身から Smibi®を使用した際の感想をスタッフへ伝えることが多くなった．感想の中には，「表情が可愛らしい，気持ちが落ちつく」など肯定的な意見が聞かれた．介入終了後には，他の SAR の購入を家族で検討するなど家族間での日常会話が増加した．また，「COVID-19 の流行が落ち着いてきたら歩いて買い物に出かけたい」と自ら目標を定め，訪問リハ介入時以外にも自主的に散歩を行うようになった．

考察

今回，訪問リハを実施している地域高齢者 1 名を対象に，コンパニオンロボットである Smibi®を緊急事態宣言が発令された 2020 年 4 月から 2020 年 5 月までの 1 ヶ月間使用し，心身機能に与える影響を調査した．その結果，Smibi®を使用することは，日内変動，睡眠，将来への絶望，不満に関する SDS スコアの改善と関連しており，心理状態の改善を認めた．

コンパニオンロボットを使用した先行研究の多くは PARO®を用いた報告である．PARO®は，アザラシ型ロボットであり，触覚，視覚，聴覚等の感覚を有し，使用者の扱い方に対してさまざまな反応を示す．Kawaguchi[17] は，21 歳から 30 歳までの 10 名の対象者に対して，PARO®を使用している際の脳血流量を functional Near-infrared Spectroscopy を用いて計測し，感情や話しかけに関わる前頭葉領域の脳血流量が増加したことを報告した．さらに，PARO®の使用でうつ病の高齢者が抱える孤独感が軽減され [18]，撫でるなどの接触の繰り返しで痛みや気分が改善される [19]．また PARO®は，対象者の抑うつ状態の改善に作用し，アニマルセラピーに近い効果を示す [20]．本研究の結果より，Smibi®のようなコンパニオンロボットとの交流を続けることで，他者との交流が少ない在宅生活においても，アニマルセラピーと同様に抑うつ性の改善につながる可能性が示唆された．

Shoesmith[21] は COVID-19 の流行に伴う隔離期間中に動物をペットとして所有していた者は，幸福感を感じる者が多く孤独感を感じる者は少なかったが，ペットが COVID-19 の感染源になる可能性があり不安を感じた者もいたと報告している．

COVID-19 の主な感染経路は，飛沫感染と接触感染が挙げられるが [22]，汚染された衣類であっても家庭での洗濯により COVID-19 を不活化することが出来る [23]．Smibi®は服を取り外して洗うことができるため，COVID-19 の感染源になるリスクは低いと考えられる．今回，Smibi®の利用者を症例と家族に限定したため，症例は緊急事態宣言が発令された期間においても安心してロボットを触ったり抱いたりすることができたと考える．

MDC に関する先行研究によると，握力は 6.11，片脚立位テストは右下肢 25.88，左下肢 26.23 と報告されている [16]．そのため，本研究における症例の握力の向上，片脚立位時間の延長といった身体機能の変化は，誤差範囲内の可能性がある．しかし，我々は Smibi®の使用により抑うつ性が改善したことに加えて，訪問リハによる屋外歩行練習やバランス練習，筋力トレーニングを通して本人の行動変容を促せたことが，身体機能の向上につながった要因の 1 つであると考える．

本研究にはいくつかの限界がある．まず，1 例報告であり 1 ヶ月間という短い期間の介入であったことである．Smibi®を用いた過去の報告は，シングルケーススタディによる報告のみである．今後は，地域在住高齢者の症例数を積み重ねることで Smibi®が高齢者の精神状態を安定させる作用があるか検証する必要がある．また過去に PARO®を使用した研究では，対象者の抑うつ状態の改善を認めるものの，ロボットの回収後には抑うつ状態が悪化した報告もある [13]．Smibi®の使用後の持続効果を確認するために，長期間の検証を行う必要がある．次に，症例はコンパニオンロボットの介入と同時に訪問リハも受けており，他者との交流を継続することが出来ていた．このことも症例の抑うつ性の改善につながった可能性がある．Smibi®の使用が本症例の抑うつ性を改善させたかを検証するためには，クロスオーバー比較試験での検討が必要であり，ロボット使用期間，ロボット不使用期間および休止期間を含め 3 ヶ月間の期間を要すると考える．今後は，長期的な視点を持って，コンパニオンロボットが COVID-19 の流行に伴う隔離期間中の人々の精神状態にどのような影響を与えるのか明らかにする必要がある．

今回，訪問リハを利用している地域在住高齢者の一症例に対して COVID-19 の流行に伴う緊急事態宣言が発令された期間にコンパニオンロボットを使用して精神状態に与える影響を調査した．結果，抑うつ状態の程度を表す SDS スコアは，37 点から 26 点に減少し，特に日内変動，睡眠，将来への絶望，不満に関する項目で 2 点から 3 点の減少となった．他者との交流が少なくなる COVID-19 の隔離期間にコンパニオンロボットとの交流を続けることは，高齢者の抑うつ性の改善につながる可

能性が示唆された．今後は，症例数を積み重ねるとともに長期的な効果の検証を行う必要がある．コンパニオンロボットの使用効果を検討していくことは，高齢者が感染対策を施しながら健康を維持するために重要であると考える．

利益相反

本論文に関して，開示すべき利益相反関連事項はない．

謝辞

本介入は，ロボット介護機器開発・導入促進事業（AMED JP：16he1202015h0004）の助成を受けた．

References

1. World Health Organization. WHO Coronavirus (COVID-19) Dashboard; 2021. <https://covid19.who.int/>. (Accessed October 9, 2021).
2. Ministry of Health, Labour and Welfare. Kokunainohasseijoukyou; 2021. (in Japanese). <https://www.mhlw.go.jp/stf/covid-19/kokunainohasseijoukyou.html>. (Accessed October 9, 2021).
3. Soni M, Gopalakrishnan R, Vaishya R, Prabu P. D-dimer level is a useful predictor for mortality in patients with COVID-19: Analysis of 483 cases. Diabetes Metab Syndr 2020; 14: 2245-2249.
4. Krendl AC, Perry BL. The Impact of Sheltering in Place During the COVID-19 Pandemic on Older Adults' Social and Mental Well-Being. J Gerontol B Psychol Sci Soc Sci 2021; 76: e53-e58.
5. Ahmed MZ, Ahmed O, Aibao Z, Hanbin S, Siyu L, Ahmad A. Epidemic of COVID-19 in China and associated Psychological Problems. Asian J Psychiatr 2020; 51:102092.
6. Philip KEJ, Polkey MI, Hopkinson NS, Steptoe A, Fancourt D. Social isolation, loneliness and physical performance in older-adults: fixed effects analyses of a cohort study. Sci Rep 2020; 10: 13908.
7. Yamada M, Kimura Y, Ishiyama D, Otobe Y, Suzuki M, Koyama S, Kikuchi T, Kusumi H, Arai H. The Influence of the COVID-19 Pandemic on Physical Activity and New Incidence of Frailty among Initially Non-Frail Older Adults in Japan: A Follow-Up Online Survey. J Nutr Health Aging. 2021; 25: 751-756.
8. Lu LC, Lan SH, Hsieh YP, Lin LY, Lan SJ, Chen JC. Effectiveness of Companion Robot Care for Dementia: A Systematic Review and Meta-Analysis. Innov Aging. 2021; 5: igab013.
9. Petersen S, Houston S, Qin H, Tague C, Studley J. The Utilization of Robotic Pets in Dementia Care. J Alzheimers Dis. 2017; 55: 569-574.
10. Folstein MF, Folstein SE, McHugh PR. "Mini-mental state". A practical method for grading the cognitive state of patients for the clinician. J Psychiatr Res 1975; 12: 189-98.
11. Togo seisakusyo corporation. Iyashigata akatyan robotto“Smibi S”; 2021. (in Japanese). <https://www.togoh.co.jp/products/care-smiby.html>. (Accessed October 13, 2021).

12. Suzumura S, Takano E, Sugishima Y, Narukawa R, Makino I, Abiko T, Oi S, Kondo I. Reduced family care burden by using a communication robot: Case report. Geriatr Gerontol Int 2020; 20: 384-385.
13. Liang A, Piroth I, Robinson H, MacDonald B, Fisher M, Nater UM, Skoluda N, Broadbent E. A Pilot Randomized Trial of a Companion Robot for People With Dementia Living in the Community. J Am Med Dir Assoc 2017; 18: 871-878.
14. ZUNG WW. A SELF-RATING DEPRESSION SCALE. Arch Gen Psychiatry 1965; 12: 63-70.
15. Keith RA, Granger CV, Hamilton BB, Sherwin FS. The functional independence measure: a new tool for rehabilitation. Adv Clin Rehabil 1987; 1: 6-18.
16. Beauchamp MK, Hao Q, Kuspinar A, D'Amore C, Scime G, Ma J, Mayhew A, Bassim C, Wolfson C, Kirkland S, Griffith L, Raina P. Reliability and Minimal Detectable Change Values for Performance-Based Measures of Physical Functioning in the Canadian Longitudinal Study on Aging. J Gerontol A Biol Sci Med Sci. 2021; 76: 2030-2038.
17. Kawaguchi Y, Wada K, Okamoto M, Tsujii T, Shibata T, Sakatani K. Investigation of brain activity after interaction with seal robot measured by fNIRS. 2012 IEEE RO-MAN: The 21st IEEE International Symposium on Robot and Human Interactive Communication. 2012: 571-576.
18. Chen SC, Moyle W, Jones C, Petsky H. A social robot intervention on depression, loneliness, and quality of life for Taiwanese older adults in long-term care. Int Psychogeriatr 2020; 32: 981-991.
19. Geva N, Uzefovsky F, Levy-Tzedek S. Touching the social robot PARO reduces pain perception and salivary oxytocin levels. Sci Rep 2020; 10: 9814.
20. Park S, Bak A, Kim S, Nam Y, Kim HS, Yoo DH, Moon M. Animal-Assisted and Pet-Robot Interventions for Ameliorating Behavioral and Psychological Symptoms of Dementia: A Systematic Review and Meta-Analysis. Biomedicines 2020; 8: 150.
21. Shoesmith E, Shahab L, Kale D, Mills DS, Reeve C, Toner P, Santos de Assis L, Ratschen E. The Influence of Human-Animal Interactions on Mental and Physical Health during the First COVID-19 Lockdown Phase in the U.K.: A Qualitative Exploration. Int J Environ Res Public Health 2021; 18: 976.
22. Mukhra R, Krishan K, Kanchan T. Possible modes of transmission of Novel coronavirus SARS-CoV-2: a review. Acta Biomed. 2020; 91: e2020036.

23. Owen L, Shivkumar M, Laird K. The Stability of Model Human Coronaviruses on Textiles in the Environment and during Health Care Laundering. mSphere. 2021; 6: e00316-21.

Figure 1 癒し型赤ちゃんコンパニオンロボット Smibi®

株式会社東郷製作所により作成されたコンパニオンロボット．本体サイズは幅200mm，奥行き 190mm，高さ 440mm であり，重量は 1.2kg である．電源にリチウムイオンバッテリーを使用し約 10 時間の使用が可能である．充電には専用のアダプターを使用する．感情表現は 500 通り存在し，使用者のあやし方により様々な反応を示す.

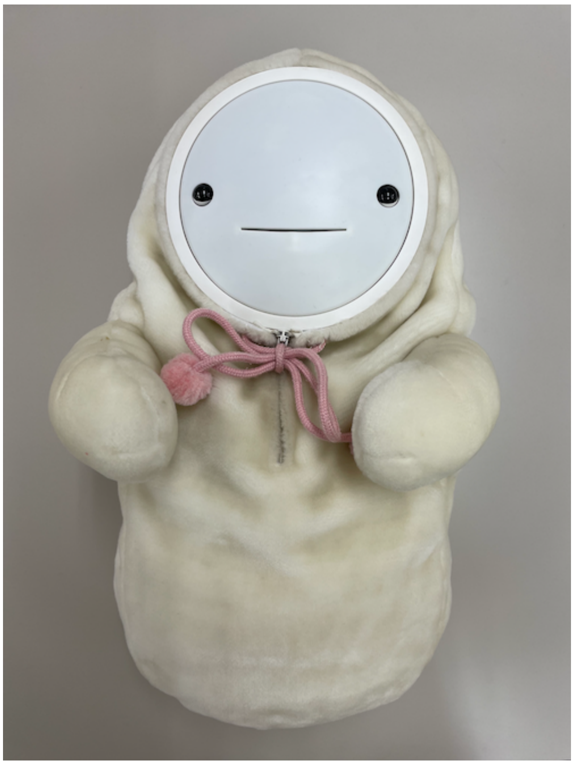

Figure 1 癒し型赤ちゃんコンパニオンロボット Smibi®

Table 1　Smibi®使用前後の評価結果

	Smibi®使用前	Smibi®使用後
握力（kg）		
Right	15.0	18.0
Left	11.9	15.0
片脚立位テスト（s）		
Right	3.0	7.0
Left	2.0	4.0
FIM (/126 points)	115	116
Motor (/91 points)	80	81
Cognitive (/35 points)	35	35
SDS (/80 points)	37	26

FIM, Functional Independence Measure; SDS, Self-rating Depression Scale.
